# Collaborative engagement with vector control stakeholders is key to enhance the utility of vector-borne disease models

**DOI:** 10.1186/s13071-025-06751-w

**Published:** 2025-04-17

**Authors:** H. E. Brown, E. Wrench, K. Wolfe, T. C. Moore, J. A. Tangena, L. Sedda

**Affiliations:** 1 Department of Epidemiology and Biostatistics, The University of Arizona, Mel and Enid Zuckerman College of Public Health, 1295 N Martin Ave, Tucson, AZ 85724 USA; 2 Lancaster Ecology and Epidemiology Group, Lancaster Medical School, Health Innovation One, Lancaster University, Sir John Fisher Drive, Lancaster, LA1 4AT UK; 3 Vector Department, Liverpool School of Tropical Medicine, Pembroke Pl, Liverpool, L3 5QA UK

**Keywords:** Vector-borne disease surveillance, Vector-borne disease control, Vector-borne disease modeling, Community-inspired science, Public health in EU and USA, Operational public health

## Abstract

**Background:**

Despite the growing complexity, computational power, and mapping capacity incorporated into vector-borne disease models, they still do not fully elucidate the role of environmental, demographic, socioeconomic, or other drivers, and rarely directly inform vector control efforts. To understand how we can improve the utility of vector-borne disease models for vector control activities, we interviewed vector control agents from the United States (USA) and the European Union.

**Methods:**

Between July and December 2023, in-depth interviews were held using a geographically targeted convenience sample with 26 individuals from organizations involved in vector control operations: 12 in the USA and 14 in the EU. We used both deductive and inductive coding of transcribed interviews to identify themes with the goal of understanding barriers to model use and uptake.

**Results:**

Despite the recognition that models could be useful, few interviewees reported that models informed surveillance and control activities, citing a mismatch in spatial and temporal scale between model outputs and operational decisions or a general lack of accessibility. Interviewees reported relying on experienced field experts and legacy protocols. Despite these critiques, there is belief that models can support operational decision-making.

**Conclusions:**

The disconnect between models and users can be improved by allowing time and resources to build collaborative relationships, by acknowledging the knowledge all members bring, and by ensuring clear communication and mutual respect. Modelers must shift their focus by aligning vector-borne disease models with operational needs.

**Graphical abstract:**

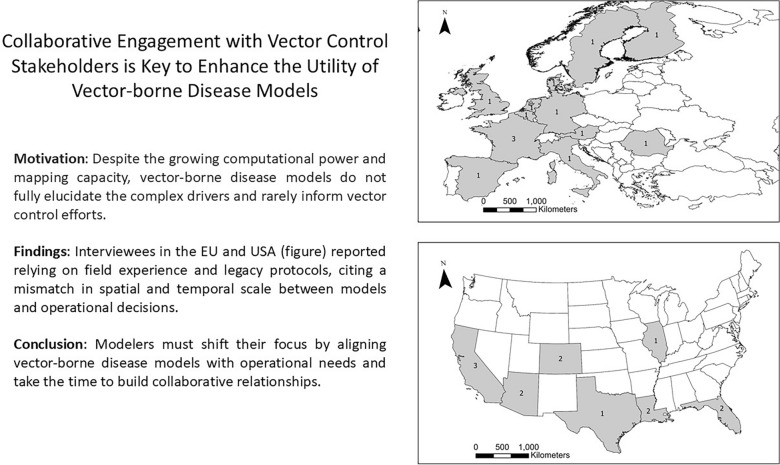

**Supplementary Information:**

The online version contains supplementary material available at 10.1186/s13071-025-06751-w.

## Background

An estimated 17% of communicable diseases globally are vector-borne (Global Vector Control Response: 2017–2030), and vector control remains the primary means to reduce disease risk [1]. However, widespread insecticide resistance, landscape modification, and emerging vector-borne disease challenges are creating a “new normal,” reinforcing the need for evidence-based vector control at a local scale and in a timely manner [2]. This includes the implementation of novel tools and strategies, learning from successful disease control programs [2] and optimizing existing surveillance strategies [3–5]. In addition, as vector-borne diseases overlap in most areas, integrating vector control across diseases and vectors is recognized as a cost-effective and successful strategy [6].

One way to address this gap is for models calibrated with surveillance data to align with operational needs. The vast majority of models built for climate-sensitive infectious diseases are for vector-borne diseases (81%), nearly half of which are focused on malaria [7]. While these models can provide valuable predictions, the inherent complexity of vector-borne disease systems makes it challenging to accurately identify specific climate, ecological, land-use, or other drivers [8]. Mounting epidemiological evidence is quantifying the negative health effects of climate change, with predictive models indicating it will get worse [9, 10].

Infectious disease models must balance the available data for parameterization and their utility for public health planning [11, 12]. In the United States (USA), vector control is conducted either within a public health institution, such as a county or state-run program, or as a mosquito abatement district, which are quasi-governmental organizations whose primary purpose is to control local mosquito populations [13]. In Europe, vector surveillance and control are generally administered at the local level (regions or local councils), with coordination at the national level. Within these systems, there are increasing efforts to compile global or regional databases to tap into the vast amount of data collected through mosquito surveillance by these institutions to support evidence-based vector control. Challenges to sharing the data include differences in methods, trapping frequency, larviciding locations, and adulticide application, which tend to be so locally specific that they vary even within a state depending on the size and funding of the program [14].

Despite the growing capacity to share data and the proliferation of vector-borne disease models (hereafter simply “models”) [15], early warning systems remain in great demand, and even models described as “operational” lack free access and user-friendly interfaces [7]. To understand the need and gaps in vector-borne disease modeling, we interviewed vector control agents from the USA and European Union (EU) and analyzed the in-depth interviews.

## Methods

### Recruitment

We aimed to interview individuals who were working within public health, academic, and research institutions involved in vector control operations. In the USA, two strategies were employed to identify interviewees. First, candidate jurisdictions were identified from a systematic review conducted by Moore et al. [16]. In brief, 24 articles with 48 co-authors from 17 departments of health or mosquito abatement districts across nine states (Arizona, California, Colorado, Connecticut, Illinois, Louisiana, New York, South Dakota, and Texas) were identified. These authors modeled human West Nile virus incidence. Second, to ensure we captured southern states where the risk for dengue incursion is greater, we searched the web to identify vector control agencies in Alabama, Louisiana, New Mexico, and Florida and identified 11 organizations/emails. Authors and representatives of vector control and public health were contacted via publicly accessible organization email addresses, organizational website messaging systems, or social media (LinkedIn). In Europe, individuals were purposefully sampled to represent EU member countries in vector-borne disease documentation such as from the European Centre for Disease Prevention and Control (ECDC), those listed as contacts on the VectorNet website, those on specific country-related vector-borne disease journal articles, and contacts between interviewees.

**Fig. 1:**
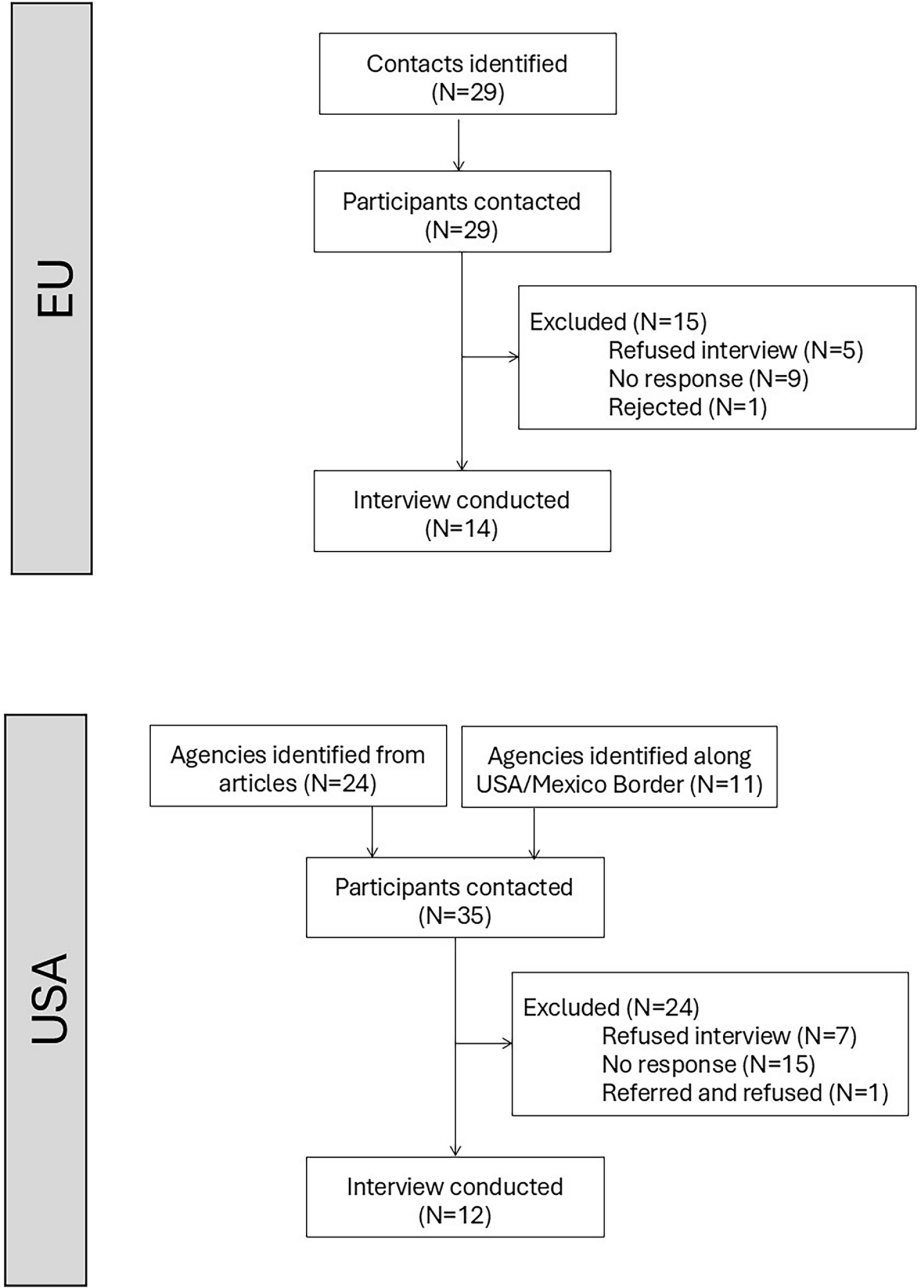
Interview recruitment flow chart, representing the number of eligible participants identified, contacted, and successfully interviewed

From June 21 to November 13, 2023, individuals were contacted up to three times, or until a representative from their country or organization responded. In Europe, out of the 29 people who were contacted, 14 were interviewed, five declined interviews due to busy schedules or no longer working in vector control, nine individuals never returned contact, and one returned contact after sufficient interviews had been carried out and themes were saturated (Fig. 1). Among the 24 US-based agencies identified through the articles, nine email requests received no response, five refused, one referred us to a different contact who refused, and nine were interviewed. Among the 11 agencies identified among the southern states, two refused, six did not respond, and three interviews were completed.

### Interviews

A total of 26 interviews were completed to understand barriers to model use and to identify factors that facilitate model uptake. An interview guide was developed prior to the interviews with a list of questions and topics to explore. Interviews were conducted and transcribed using Zoom (Zoom Video Communications, Inc., San Jose, CA) or Teams (Microsoft Corp., Redmond, WA). Even though the study was deemed exempt, participants were asked to verbally agree to the recording and transcription and were reminded that they could end the interview at any time and their recordings would be removed. Transcription texts were cleaned to correct transcription errors and then imported into NVivo (Lumivero, Denver, CO) for subsequent analysis.

### Data analysis

We used a grounded theory approach whereby data collection and analysis inform one another, and the data are grouped into categories or themes [17]. We used both deductive and inductive coding. The goal of the project was to understand barriers to model use and to identify factors that facilitate model uptake (deductive codes). Inductive codes emerged from the transcript review. The Lancaster team met to identify a list of candidate codes. These were shared with the UArizona team for additional code identification and for code validation. Once the initial codes were agreed upon, the codebook was shared.

Investigators familiarized themselves with the transcript data through repeated reading. Each transcript was independently coded line-by-line by two investigators using the codebook (with the goal of each transcript being coded by one participant who conducted the interview and one who did not). Early in the coding, the two coders compared and refined the conceptual coding categories. Once transcripts were coded, the two principal investigators (PIs; LS, HEB) met to discuss connections between the codes until a final agreed-upon set of thematic codes was identified. Themes related to modeling choice, use, and needs are described here.

To ensure that we did not inadvertently identify participants or grossly misrepresent the conversations, a draft of this manuscript was shared with and approved by all participants.

## Results

### Sample characteristics

**Fig. 2:**
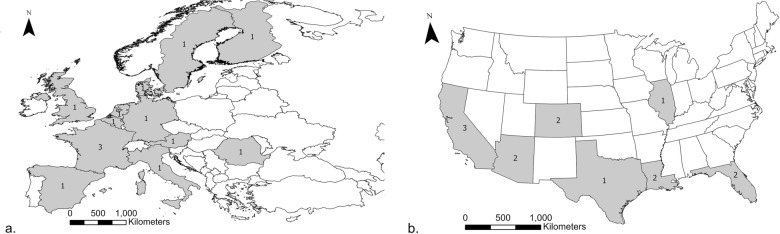
Visualization of the study participants and their locations. EU countries (**a**) and USA states (**b**) where interviewees are based are shown in dark with the value denoting the number of representatives interviewed

Between July 3, 2023, and December 12, 2023, 26 members of public health, academic, and research organizations were interviewed, 14 in the EU and 12 in the USA (Fig. 2). When possible, interviews included two interviewers, but time zones and availability were sometimes restrictive (10/26 (38.5%) interviews had only one interviewer). Individuals interviewed were primarily working within health organizations (*n* = 11 departments of health or public health: seven EU, four USA) or vector control organizations (*n* = 10; three EU, seven USA), with four organizations focused primarily on research and one on parks and recreation. Interviews lasted 49 min on average (min: 17, max: 88). When noted, most interviewees had PhD degrees (*n* = 13) and had been in their position for an average of 19 years (min: 4, max: 38). About half of the organizations described themselves as small (< 10 full-time positions, *n* = 12), and many reported seasonal employees that doubled the number of staff. Most organizations that did not themselves engage in research reported liaising with academic institutions (15 of 20).

### Defining a model and model uptake

The term “model” is multifaceted, and interviewees interpreted the word in diverse ways, reflecting their different operational and research perspectives. For example, several USA vector control agencies used “model” to encompass calculating the vector index or mapping disease or vector hotspots. For some, a model might represent a mathematical framework to predict disease spread, while for others it could represent an empirical tool designed for intervention strategies.

Ecological niche models were identified as useful for estimating where to survey, based on existing data. Descriptive analyses were used to classify traps based on vector diversity and phenological factors (EU), and abundance modeling was mentioned to understand what was driving vector spread or their presence/absence. However, the theoretical usefulness of models struck with the reality of operational public health and questions about model quality and utility started to enter the conversation.

*… abundance modeling, … that is never gonna work, because the spatial–temporal variation is so high that … it's not informative or gives at least the wrong information. —EU*

Even if not directly the goal of a risk model, models that could help restrict cost were discussed, for example, models to help optimize the number and frequency of surveillance locations and collections to make it cost-efficient (e.g., “good data with minimum workload/cost” —EU).

Vector control representatives recognize that vector-borne diseases are complex, leading to recognition of a need for integrated modeling. In other words, models should not be designed in isolation to address a single concern; rather, they need to address surveillance, plus public health and economic effects. Translating that complexity to decision-making is another layer of challenge.

*Even us, who have been working with it for years, find it very difficult sometimes to figure out what the transmission potential is. —EU*

As a result, models are generally just taken as an added piece to the vector-borne disease puzzle but not viewed as particularly useful. Lack of uptake is partly attributed to the model output not being designed with application in mind. Thus, rather than models, fellow vector control representatives and partners were valued for sharing the design and for tracking vector control.

### Themes

We identified six themes with respect to obstacles to model uptake. We focused on why models are not used, why they are not useful, and why they do not provide anything new. Additionally, we focused on what kind of models the interviewees would be interested in, examples of when models do work, and overall challenges in achieving higher utilization of models in vector control.

#### “We don’t use models or do modeling”

Despite the recognition that models might be useful and collaboration with modelers is common, many reported not actually using the models. Reasons included that models did not appropriately inform their surveillance and control operations, or even case prediction/preparedness. Additionally, models were believed to be not easily accessible, as they were not publicly accessible or were developed without an easy user interface.

*We just got to figure out a way to make that stuff accessible, so that people can make really good decisions. —USA*

The time investment to fully engage with models as well as the challenges of incorporating all the complexities of vector control were cited as factors which make model development within their own institution unfeasible. Even though some interviewees recognized their capacity for building their own models, carving out staff time is a restricting factor.

*Yes, that’s a big time issue, to spend a lot of time on that when you have inspections to do and complaints and you start getting 20 complaints a day for each staff member, you know, it's kind of hard to justify spending a lot of time on something like that. —USA*

Given the time and mission focus on vector control and surveillance activities, it is easier and more efficient to collaborate with external experts, often academics. This not only allows vector control to focus on its mission but grows the reach and success of vector surveillance activities.

#### “Models aren’t useful”

Although the possible utility of models is evident, models that the interviewees were aware of were not useful for their day-to-day activities in designing and implementing control activities. The greatest critique for models was that they are not sufficiently real-time and that they do not match the scale of operational decisions and interventions.

*When we are implementing a vector control, we do it on a radius of 150-200 meters. —EU*

*Modeling has a role, but not on a day-to-day operational scale at this point in time. —USA*

*They are of no use to us in our daily decision-making if they come out once a month … You gotta match the granularity, and at resolution time and space, with the granularity of how those decisions are made. —USA*

Overwhelmingly, there was an acknowledgment that most models built with vector control data did not result in operationally useful models.

*…academic collaborators that come in and give us a little bit of insight … It's useful, but to date we've not worked with a collaborator that's then given us some operationally useful model. —USA*

With a strong desire to ensure the models are clear and ethical:

*…figure out a way to make that stuff accessible so that people can make really good decisions that are in alignment with their community’s ethics. —USA.*

*…But I don’t remember having seen any clear paper showing that this kind of model will help you to or will help you just to design your controller actions. —EU*

The lack of operationally useful models has supported the growth of in-house thresholds or risk systems based on simple and pragmatic approaches. For example, one vector control reported using a very simple classification of risk by taking as many environmental components as possible, scoring them on a level from 1 to 5 and generating an average that could then be associated with observed human cases. The complexity, inaccessibility, and limited applicability of existing models are driving organizations to create informal alternatives, while models may offer greater robustness and reliability, and validity.

#### “Models don’t tell us anything we don’t already know”

Even with the high staff turnover experienced by some agencies, day-to-day operational decisions often rely on field experience. Decision-making relied on national guidelines (e.g., from the Centers for Disease Control and Prevention [CDC] and ECDC), established practices, or observations from the field—by looking at the data and operational thresholds. As models do not further enhance the result, they are not being used.

*And then some of the results are kind of just common sense for field experts. —USA*

*… We've got a pretty good idea of what's going on, pretty good indications of when activity is going to peak and what climate fluctuations are doing. So you just have to outperform what we already have. —USA*

Furthermore, the current lack of efficient early warning system models leaves vector control and public health reactive—waiting for and responding to the first cases.

*Then the conclusion was then we have to wait for the first person, but that's not very early warning. —EU*

However, with landscapes rapidly changing due to urbanization and deforestation, incursions of new vectors, and changes in weather patterns, the applicability of field experience may be reduced.

#### "What I want from a model"

Despite the critiques of models, there is a desire to find ways to make them more helpful. There is overall confidence that modeling can support vector control operations.

*I think there is some modeling that could help us, I'm sure out there. —USA*

With busy vector control agents, automation is critical. Regardless of the eventual output, vector control users want something very integrated, where users just “…add [their] data to it and it will sort of run itself” (USA). The lack thereof is also cited as a barrier to model uptake.

*One of the reasons why we have not used modeling is because it's not quick. We can’t apply it. —USA*

Potentially useful models include ones that can be “run every day, every morning …[yielding] a heat map … [of] the problem spots …” (USA). Ideally, models that are early-warning and allow for intervention “… at the appropriate time and early in the transmission cycle to reduce risk for the populations in which we were responsible for is a benefit” (USA).

One suggestion was to rethink models from predicting trap counts to helping to prioritize vector control, and to quantify the efficiency of the control, for example, using models to assess the efficiency of the different control efforts or models of control (truck, spray, aircraft). Rather than predictive models, models should be designed to support planning or designed and used to play out and choose between various surveillance and control scenarios, which then directly feed into operational decisions “like upgrading or downgrading depending on the scenario that you are” (EU).

*We need source-based modeling to help us define where we spray. —USA*

Future models need to include insecticide resistance factors, to predict negatives (to explain not only why counts are high, but also why they are not), and to address more of the biology that might be driving spatial and temporal vector dynamics. But, for models to be integrated into modeling and surveillance programs, they have to be simple and easy.

*The best model is a simple model. … Having something that is very simple and very accurate, that's the dream. —USA*

*[The model] has to be easy to use and concrete. —EU*

#### When models work

Models have been successfully integrated when there has been collaboration between modelers and vector control representatives to integrate observations with ecology and biology (e.g., temperature to augment vector index thresholds). Vector control representatives recognize the need to share the data and to take the time to ensure the data are understood.

*So, that is something, unless you collect your own data, when it comes to field work, you don’t understand all the different vagaries that go into what that data is and honestly just how complicated mosquito control can be. —USA*

The importance of good data was acknowledged as vital for good models. This included acknowledgment that there is a considerable amount of data housed at vector control agencies.

*… if we want to have a good model output, we have to collect the data in the right way at the right place and etcetera. —EU*"

*… we don’t have the time to collect all the environmental data to put in a model… It just hasn’t been helpful for us to use a model because of that. —USA*

Sharing the data with interns and graduate students is one way to expand the network of collaborators. However, depending on the experience and supervision of the students, the products can be “pretty basic.”

### Challenges which may influence model integration

Staff recruitment, including candidates with the necessary expertise, and high turnover create challenges to maintaining vector control efforts. This lack of human and financial resources serves primarily to limit the collection of sufficient data. Through collaboration, existing expertise, and prioritization of effort, interviewees noted that the capacity deficit did not prohibit using collected data or sometimes even expanding capacity for specific projects (e.g., taxonomy). It is recognized that increasingly there are vector and vector-borne disease experts coming out of universities which will bolster existing expert capacity. Furthermore, community members are increasingly engaging in vector surveillance and sometimes even control, which can further expand capacity.

The lack of resources is compounded by a general lack of coordination between sectors and minimal automatization in surveillance. So, while vectors have no borders, the data do. Collaborations can support planning (e.g., vector control and public health actors co-participating in workshops to plan for impending incursion of West Nile virus [WNV]) and sometimes even the realization that plans need to be developed because sectors might soon have increased vector control responsibilities.

General community awareness can be a limitation as well. In fact, communities where infectious disease is not as extensive may be less aware of the risks and more reluctant to support vector control efforts and biocide use. This can limit data collection and subsequent modeling efforts. Proactive engagement with the public and transparency in spraying yield greater appreciation of the vector control work that is being done. Key to the success was packaging of information so communities could digest and understand it and feel confident that they could trust it.

## Discussion

Using a convenience sample, we conducted 26 semi-structured interviews among vector control organizations. Most interviewees were from health organizations, had PhDs, and had been in their position for more than a decade. Most did not engage in modeling themselves; rather, they liaised with academic institutions for support. We identified themes around challenges to model uptake, grouped by barriers for use, what models do, and what the gaps are for vector-borne disease modeling.

### Limitations 

This is a qualitative study designed to explore challenges to adopting models for vector control. Although we originally identified 35 USA and 29 European organizations, our response rates—34.3% and 48.3%, respectively—were comparable to what others have found for executives/top managers, and we covered our intended geography. Nonetheless, we did not comprehensively sample vector control agencies, and there is likely additional variation not captured. We recruited individuals using different strategies (i.e., based on co-authoring or based on geography). However, we did not detect any trends with respect to academic leanings: those identified through geography also published in academic journals, and almost all reported liaising with academic institutions. This reinforces the notion that robust and bidirectional relationships are critical to successful integration of models. Finally, given the various definitions of model and possible interpretations of “useful,” it is not feasible to stratify and compare between those we might identify as users versus non-users. This paper is thus an exploration into revising the vector control modeling agenda. Specific exploration of examples of successful integration might elucidate components facilitating uptake.

### Making stakeholders partners

Challenges with respect to the capacity for building models in either hiring contractors or in-house skills was identified in our interviews as a limiting factor in the development and uptake of models, as it had been in prior studies [18]. Those agencies who partnered with academic institutions or were able to build models in-house did report positive views of models.

How stakeholders are engaged by research teams may influence model uptake. Gerlak et al. found that in studies with stakeholder engagement, 95% of instances of engagement involved taking surveys or sharing collected field data, i.e., as data generators not partners [19]. They showed that research findings are rarely disseminated to the stakeholders (23%) or the stakeholders are rarely active participants in research agenda prioritization or data analysis. This may stem from a difference in mission: Public health, vector control and modelers generally share the same mission of protecting health, while the means, accountability, and priorities differ. For example, vector control space spraying activities prevent diseases, while simultaneously controlling nuisance species—it therefore does not require regular surveillance if the end result is to remove the nuisance. However, from a modeling standpoint, tracking changes in vector dynamics requires regular surveillance of such locations to make the intervention cost-effective.

These mission differences can create challenges to working together. Immersive experiences with reciprocal embedding of academics and stakeholders can lead to more robust co-production processes and dialogue as a valued output [19]. Ferguson et al. suggest three actions to patch the disconnect between models and users: (1) allowing time for collaborative relationships to mature though sincere and respectful interaction, (2) recognizing the contextual knowledge all members of a team bring, and (3) attending to the engagement effort to ensure clear communication and mutual respect [20]. We found similar feedback from the interviewees.

### Modeling to the need not the data

Vector agencies are coming together to share data [13]. As journals require the publication of data, they too are becoming a source for vector data (e.g., https://www.gbif.org/) [21]. The efforts of citizen scientists can further augment the collection of valuable vector data [22, 23]. This increased accessibility of data, in combination with increased computational capacity and changes in vector-borne disease risk are leading to the proliferation of models to predict changes in vector-borne disease [15]. Yet, although they are built using vector-borne disease surveillance data, participants in this study found that existing models do not meet their vector control needs.

Vector control is time- and resource-intensive, with resistance threatening its effectiveness [24]. Existing surveillance data can be used to design ecological sampling that optimizes trapping location and frequency, balancing effort with data quality [3]. Participants in this study repeatedly asked for models that inform the daily vector control efforts in a user-friendly format which requires little effort to import live data and implement the model. If the goal of vector control and of disease modeling is ultimately to prevent vector-borne diseases, then modelers must shift their focus from model creation to useful model development. Establishing and maintaining relationships between modelers and vector control can support clarity in what the modeling needs are and how they can be achieved to close the model use gap [25]. One tactic we have employed to allow for engagement constraints (see Challenges theme) is through the academic classroom—with vector control agencies serving as “clients” for the classroom, sharing the data and shaping the conversations, while informing expert modelers.

## Conclusions

We found that a gap remains in vector-borne disease modeling, where those who collect the data are not benefiting from the modeling of their data. We maintain that a shift in ethos from viewing vector control as a data source to a model client might result in the development of models that are useful to vector control, in particular by being timely and fitting with the required spatial resolution. Further, useful models can reduce disease burden by optimizing vector control, and closer collaboration can result in better data with novel uses. Truly useful models, however, will only come with robust, bidirectional collaboration.

## Supplementary Information

Supplementary material 1.

## Data Availability

The data supporting the main conclusions of this study are included in the manuscript. Qualitative data used and/or analyzed during the current study can be obtained from the corresponding author upon reasonable request.

## Abbreviations

CDC: Centers for Disease Control and Prevention
ECDC: European Centre for Disease Prevention and Control
EU: European Union
USA: United States
