# Supplementary material for: Collaborative engagement with vector control stakeholders is key to enhance the utility of vector-borne disease models
Source: Parasit Vectors. 2025 Apr 17;18:143. doi: 10.1186/s13071-025-06751-w (PMC12007198; doi:10.1186/s13071-025-06751-w)
Supplement: Supplementary file 1 — Supplementary material 1. [file 13071_2025_6751_MOESM1_ESM.docx]

This is the question guidance document. This is meant to guide the conversation, but not each question needs to be asked if it was already answered. It is also meant to be adapted depending on the person being interviewed.

The “[]” content is to remind the interviewer of the project aims, but doesn’t need to be read aloud.

# Framing

Thank you for your time in speaking with me today. We wanted to talk with you today because you are a coauthor on a manuscript or public health manager/head of division that uses VBD models and your affiliation is vector control. The goal of our project is to understand how models are being incorporated into West Nile virus or other emergent vector-borne diseases (Chikungunya) planning and control.

What flagged you for us was:

{INSERT CITATION/ REASON HERE}

We have about 13-20 questions (depending on if you count subparts), and if it is ok I would like to record the audio for transcription purposes. Would it be ok for me to start that now?

[If co-author on paper]

1. That paper was published in {YEAR}. Can you tell me a little about your role on the paper?

[Potential Probing Questions:]

[To understand depth of data handover/explanation] Was your role providing data? If so, how did you collect the data and can you tell us about the handover?

[To understand depth of engagement by modelling team] Did the lead authors talk with you about vector control in your area?

[If manager/other not reached because of publication]

1. Can you tell me a little about your role in the public health institution?

[Potential Probing Questions:]

How do you organize the day-by-day vector intelligence or control activities?

Do you have daily alerts?

# Context Questions

These next questions are to help me understand what Vector Control looks like in your jurisdiction?

1. How long have you personally worked in vector control?

___ years

1. Would you say VC and planning for VC is a large part of your organization’s role? Are there dedicated “VC” departments/staff?
2. What are the kinds of activities your organization does?
   1. [Spraying/ Larvacide / Surveillance trapping]
   2. Frequency?
   3. Length of Season?
   4. Do you also issue alerts/ public messaging?
      1. If so what triggers them?
3. Do you use digital tools to collect and analyze the surveillance data you collect?
   1. If yes, can you tell me a little about those?

# Main Questions

Thank you for helping me to get an understanding of vector control in your region. [For authors: We started with the publication which you were a part of using vector control data to build a predictive model of WVN in your area]. These next questions are specifically about the use of predictive modeling in planning vector control.

1. In what way do current advances in research and expert opinion influence your vector surveillance strategies?
2. To your knowledge, did the model get used to change any WNV control strategies?
   1. If not, do you have any thoughts as to why not?
3. Do you use/ work with modelers in your planning of vector surveillance?
   1. If yes, can you tell me a little about those?
      1. What kind of models?
      2. Who builds them (e.g., in-house, academic partners)?
         1. [To understand motivation/resources] Was there a reason it isn’t done in-house?
4. If you work with modelers,
   1. [If co-author on paper: can you tell me about whether, and if so how working on that paper influenced your thinking about your surveillance sampling strategy? ]
   2. Did working with modelers give you insight into how the data you collect are being used?
   3. Did working with modelers influence you to change the frequency or locations of trapping?
5. What do you think might increase the integration of modeling in VC programs?
   1. [What facilitated/obstructed applying models to VC]

# Exploratory Questions

These final questions are again broader about vector control and the challenges to vector control.

1. What challenges do you experience when thinking about and planning for the vector control season?
   1. Can you describe a big success or a big “fail” you experienced working in VC?
      1. [Do you think a model could have helped?]
2. Do you have a ‘wish’ for when it comes to vector control?
   1. [What time scale]
   2. [What spatial scale]
   3. [With what predictive accuracy? i.e., what do you want to predict?]

# Wrap-Up

I appreciate that you took the time to talk with me today to help me understand vector control from your perspective and the use of models in vector control.

1. Is there anything you wish I had asked, anything you’d want researchers to know about vector control how can be improved?
